# Preparation and characterization of a novel biodegradable film based on sulfated polysaccharide extracted from seaweed *Ulva intestinalis*

**DOI:** 10.1002/fsn3.2370

**Published:** 2021-06-28

**Authors:** Mohammad Nabi Davoodi, Jafar M. Milani, Reza Farahmandfar

**Affiliations:** ^1^ Department of Food Science and Technology Sari Agricultural Sciences and Natural Resources University Sari, Mazandaran Iran

**Keywords:** Biodegradable film, Glycerol, Polyethylene glycol, Seaweed, Sulfated polysaccharides

## Abstract

Seaweeds can be a suitable, inexpensive, abundant, and renewable source for the production of biodegradable films as an alternative to plastics. Sulfated polysaccharides, which are abundant in *Ulva intestinalis* seaweed, have shown important biological activities such as anticoagulant, antioxidant, antitumor, anti‐inflammatory, and antiviral activities. Mechanical, physicochemical, barrier, and surface properties of sulfated polysaccharide films extracted from *Ulva intestinalis* using glycerol and polyethylene glycol (PEG) as plasticizers were studied. *Ulva intestinalis* sulfated polysaccharide films (USP films) were successfully prepared by the incorporation of three concentrations of plasticizers (30, 40, and 50%). The film properties depended on the type and concentration of the plasticizer. Based on the results, by increasing the concentration of the plasticizer, the thickness, moisture content, solubility, and elongation at break of the USP films increased and tensile strength, young's modulus, transparency, and barrier properties of the films decreased. The film plasticized with 30% PEG showed the highest value of tensile strength (36.95 MPa), and the lowest value for permeability to vapor water and oxygen were 1.9 g mm^‐1^ s^‐1^kPa^‐1^ × 10^–11^ and 7.45 cm^‐3^.cm/cm^2^.s.cmHg ×10^–8^, respectively. Scanning electron microscopy (*SEM*) observations indicated that the surface of the films was free of bubbles, cracks, or fractures. Fourier transform infrared (FTIR) spectroscopy results revealed some interactions between plasticizers and the polymer.

## Graphical abstract

Ulva Intestinalis could be a new source for edible film production, nevertheless the mechanical, optical, physical, and barrier propertiesof the films were a function of type and concentration of plastisizer.

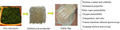

## 1 INTRODUCTION

In recent decades, synthetic polymers (plastics) have gradually taken over the packaging market owing to their lower price, better durability, and excellent waterproof performance. Most of these plastic packages are non‐degradable and considerably take more than 100 years to decompose, raising food safety concerns and environmental pollution issues (Briassoulis and Giannoulis, 2018; Eygen et al., 2017). Currently, between 22% and 43% of the world's plastic waste is disposed of by landfilling, which leads to a negative effect on the environment (Van Eygen et al., 2017). Therefore, it is necessary to replace them with biodegradable polymers that have biodegradability and biocompatibility in the environment. Various biopolymers have been applied for this purpose, the most common of which are polysaccharides (Feki et al., 2019), proteins (Piccirilli et al., 2019), and lipids (Gonçalves et al., 2019).

In the food industry, edible films are a potential alternative to plastics and can be served to reduce the negative effects of plastics on the environment, and much research has been done on the use of film ingredients such as the use of pectin (Da Silva et al., 2019), gelatin (Díaz‐Calderón et al., 2018), chitosan (Nataraj et al., 2018), and starch (Dai et al., 2019). Polysaccharide films are shown good mechanical properties, nonetheless they are highly permeable to water owing to of hydrophilic nature (Seyedi et al., 2014) and brittle due to extensive interactions between polymer chains through hydrogen bonding, electrostatic forces, hydrophobic bonding, and cross‐linking (Haq et al., 2014). The production of edible films requires plasticizers to blend with the polysaccharides to increase their workability and flexibility by decreasing intermolecular forces and increasing the mobility of polymer chains. Correspondingly, they can improve the mechanical and barrier performance of the films at different degrees (Zhang et al., 2016). Glycerol, polyethylene glycol, sorbitol, and other polyols have been widely applied in the manufacture of edible films as plasticizers (Cao et al., 2018).

Recently, seaweeds have attracted much attention in various fields, such as energy, food, and even medicine. Seaweeds are plant‐like organisms that generally live attached to rocks or attached to other hard infrastructure near coastal areas. They are generally classified as red seaweeds (such as *Rhodophyte*), brown seaweeds (such as *faecalite*), and green seaweeds (such as *chlorophyte*) (Khalil et al., 2017). The excellent carbohydrate content of seaweeds has led to the industrial use of its species as a source of hydrocolloids (derivatives of seaweed), such as alginates, carrageenans, and agar in the fields of food technology, biotechnology, microbiology, and medicine (Khalil et al., 2017). These hydrocolloids are defined as long‐chain polymers (polysaccharides) characterized by their ability to form gels when dispersed in water.

Sulfated polysaccharides are bioactive macromolecules in which some of the hydroxyl groups of the sugar residues are substituted by sulfate groups. *Ulva intestinalis* is a green seaweed belonging to the *Ulvaceae* family that is composed of intertwined filaments. They are a rich source of sulfated polysaccharides located within the intercellular space and the fibrillar walls (Wang et al., 2014). This type of polysaccharides has shown important biological activities such as anticoagulant, antioxidant, anti‐tumor, anti‐inflammatory, and anti‐viral activities (Peasura et al., 2015).

This research was designed to characterize the *Ulva intestinalis*–sulfated polysaccharide (USP) films prepared using different types of plasticizers at various concentrations.

## 2 MATERIALS AND METHODS

### 2.1 Materials

The green seaweed *Ulva intestinalis* was collected from the coast of Farahabad, Mazandaran province, Iran, in September 2019. Then, the fresh seaweeds were washed using seawater, followed by tap water, and dried at 60℃. The dried seaweeds were pulverized with a blender, sieved (<0.4mm), and stored in a plastic bag at −20℃. Glycerol, polyethylene glycol 600, acetone, and ethanol of 96% purity were purchased from Merck Corporation (Readington Township, NJ, USA).

### 2.2 Extraction of sulfated polysaccharides

Briefly, 200 g of the milled seaweed was treated with 2 L ethanol (80% w/v) under constant mechanical stirring overnight at room temperature to remove pigments, lipids, some phenols, and low molecular weight compounds. To separate the sediment from the ethanol solvent, a refrigerated centrifuge with the controlled temperature at 10℃; 8,000 rpm for 10 min was applied, and then the supernatant was discarded. The residual was rewashed with EtOH (80% w/v), rinsed with acetone, centrifuged at 10℃ and 10,000 rpm for 10 min again, and dried at room temperature in a fume hood.

To extract the polysaccharides, 40 g of depigmented powder was added to 1 L distilled water and the extraction was carried out at 65℃ with a stirrer for 2 hr. The supernatant was collected after centrifugation at 10℃ and 10,000 rpm for 10 min, and the extraction was conducted twice. The supernatant was concentrated using the rotary evaporator under reduced pressure at 60℃. Eventually, the concentrated extract was frozen at −20℃ and placed in a freeze dryer, and then pure USP powder was obtained (Tab arsa et al., 2018).

### 2.3 Film preparation

Aqueous solutions were prepared using the method described by Carneiro et al. (2009) with some modifications. The USP was dissolved in distilled water (1.5% w/v) with a magnetic stirrer at 200 rpm for 24 hr at room temperature. Glycerol and PEG were added as plasticizers at the same concentrations in the range of 30, 40, and 50% USP weight basis; also Tween 80 at concentration 0.1% was subsequently dissolved in this solution. Finally, 60 ml of each solutions was poured into a circular Teflon dish with a diameter of 16 cm and dried at 35℃ for 16 hr. The obtained films were conditioned in a desiccator at 25 ± 1℃ and 50 ± 1% RH for two days.

### 2.4 Film thickness

The thickness of the films was measured by a manual micrometer (Helios Preisser model 0850, Germany) with a precision of ±0.01 mm at ten random locations of each film. Measurements were replicated four times with independent film samples, and the mean values were reported.

### 2.5 Mechanical properties

Mechanical properties were measured with a texture analyzer (CTX, Brookfield, USA), and tensile strength (TS), Young's modulus (YM), and elongation at break (EB) were determined according to ASTM D882 (ASTM, 2001) with some modifications. Once the mechanical test, the films were cut into stripes (1 × 10 cm) and were conditioned at 25℃ and 50% RH for two days. The thickness of each film was calculated (measuring at ten different points), and their mean was taken as the sample thickness. The initial grip separation and crosshead speed were set at 50 mm and 10 mm/min, respectively. TS and E of the films were calculated by Equations (1) and (2), respectively:

(1) $$\mathrm{TS}=\frac{F}{L\times X}$$

(2) $$E\%=100\times \frac{A‐A1}{A1}$$

where *F* is the tensile force (*N*), *L* the width of the film (mm), *X* the thickness (mm), *A*_1_ is the initial length of the film, and *A* is the length of the film at breaking point. Five consecutive readings were performed for each film.

### 2.6 Moisture content and solubility

Moisture content (MC) of the films was measured by the method described by Sahraee et al. (2017) in which 2 × 2 cm^2^ pieces of the films were dehydrated in an oven at 105℃ for 24 hr. The measured difference in the sample weight before and after oven‐drying was reported as moisture content.

To obtain water solubility of the films, three pieces of oven‐dried films were immersed in 50 ml distilled water and held for 24 hr with gentle shaking using a magnetic stirrer at 200 rpm. Then, the samples were filtered through a filter paper and dried at 105℃ for 24 hr. Subsequently, the solubility of the films was calculated by:

(3) $$S\%=\frac{m1‐m2}{m1}\times 100$$

where *S* is the solubility of the film, m_1_ is the initial weight of dry film before immersing in water, and m_2_ is the final weight of dried films. This experiment was performed in triplicate.

### 2.7 Water vapor permeability (WVP)

The WVP of USP films were calculated according to the ASTM standard test method E96/96 M (ASTM, 2015) with some modifications. Glass cups (with a depth of 100 mm, and an internal diameter of 20 mm) were preconditioned at 105℃ for 24 hr to remove any water. The cups were filled with calcium chloride at half the bottle volume to reduce their internal RH to 0%. The film samples were fixed on the glass cups with paraffin as a sealant. The glass cups were placed in a desiccator containing a saturated solution of NaCl to provide 75% relative humidity, at 25℃. Weights of the glass cups were recorded in regular intervals (24 hr) with 0.0001 g accuracy for two weeks, and WVP was calculated by:

(4) WVP=w/t×x/Δp×A

where w/t is calculated by linear regression (*R* ^2^ > 0.99) from water absorbed by the system when the steady state was reached, x is the average film thickness, A is the film area exposed to moisture transfer, and ∆p (kPa) is the difference of vapor pressure between the inside and outside of the glass cups, which is calculated by:

(5) ΔP=S×(R1‐R2)

where *S* is the saturated vapor pressure at 25℃ (3,166 kPa), R_1_ and R_2_ are the relative humidities in the desiccator (0.75) and the interior of the cups (0), respectively.

### 2.8 Oxygen permeability

Oxygen permeability (OP) of the films was measured with a gas permeability tester (GTR, Tehran, Iran) according to the ASTM D standard test method (ASTM, 2002). A sample film was thus sealed between two chambers, each one with two channels one for gas inlet and the other for the gas outlet. Oxygen was supplied at a controlled flow rate in the lower chamber, with an electronic flow meter to keep the pressure constant inside that compartment. The other chamber was purged by a stream of nitrogen, similarly at a controlled flow rate; nitrogen acted as a carrier for oxygen, and oxygen permeation values were determined using chromatography.

### 2.9 Transparency

Transparency of the films was measured according to the method of Gontard et al. (1992). USP films were cut into rectangular strips of 1 × 4 cm and placed in the spectrophotometer cell. The empty cell was considered as the control, and the spectrum of each films was recorded using a UV–Vis spectrophotometer (PG instrument, UK). The relative transparency of the films was measured at 600 nm, and transparency values were obtained by:

(6) $$T\;=\;\frac{A600}{X}$$

where *T* is transparency, *A* is the absorption rate at 600 nm, and *X* is the film thickness in mm.

### 2.10 Film color

To investigate the effect of plasticizer content on the color of USP films, the films were evaluated with a colorimeter (IMG Pardazesh, Iran). A black box with dimensions of 50 × 50 × 50 cm with a white background was employed as the imaging platform. The lighting system consisted of two 10‐watt fluorescent lamps, each about 40 cm long, located in the middle of the shooting room. Digital imaging was performed at a distance of 20 cm from the samples. Images were saved in jpg format and RGB color format, and images were converted to L*, a*, and b* using Image J software and Color‐Space‐Converter application.

### 2.11 Fourier transform infrared spectroscopy (FTIR)

The FTIR spectrum was measured with a spectrophotometer (Agilent Cary 360, USA) in the region of 400–4000 cm^‐1^ and by 40 scans at a resolution of 4 cm^‐1^ (Thakur et al., 2016).

### 2.12 Scanning electron microscopy (*SEM*)

Morphology of the USP films was observed using a scanning electron microscope (SNE4500 M; SEC Co., Ltd.) under an accelerating voltage of 20 kV. USP films were coated with gold by a sputtering system (MCM‐100; SEC Co., Ltd). The films were observed at a magnification of 1,000×.

### 2.13 Statistical analysis

The experimental data were analyzed by the factorial method of SAS software version 9.1 (SAS Inc). To compare differences among data, analysis of variance (ANOVA) was employed, and Duncan's multiple range test was then used to establish differences among treatments at a 5% level. Excel 2016 software was used to draw the relevant curves.

## 3 RESULTS AND DISCUSSION

### 3.1 Thickness

The effect of different types and concentrations of plasticizers on the thickness of USP films are shown in Table 1. The thickness of plasticized films increased significantly (*p* ≤.05) with the increase in plasticizer concentration regardless of their type. The increased thickness of USP films by incorporation of the plasticizer can be described by their function in disturbing the intermolecular bonds between polymer chains, which leads to rearrangement of the polymer configuration to a more expanded structure and a thicker polymer film (Razavi et al., 2015).

**TABLE 1:** Effect of plasticizer type and concentration on tensile strength of USP films

Film type	Thickness (mm)	TS (MPa)	YM (MPa)	E (%)
USP +30% Glycerol	0.041 ± 0.002^e^	20.25 ± 0.04^b^	852.62 ± 0.42^b^	19.49 ± 0.52^d^
USP +40% Glycerol	0.06 ± 0.001^c^	12.15 ± 0.01^d^	232.97 ± 0.12^e^	25.72 ± 0.46^b^
USP +50% Glycerol	0.071 ± 0.001^b^	5.91 ± 0.04^f^	75.44 ± 0.07^f^	38.79 ± 0.6^a^
USP +30% PEG 600	0.049 ± 0.001^d^	36.95 ± 0.03^a^	1,048.36 ± 1.1^a^	11.79 ± 0.33^f^
USP +40% PEG 600	0.07 ± 0.001^b^	16.17 ± 0.01^c^	508.02 ± 1.2^c^	18.07 ± 0.59^e^
USP +50% PEG 600	0.083 ± 0.002^a^	11.24 ± 0.01^e^	283.72 ± 0.36^d^	24.4 ± 0.63^c^

Note

Values are given as mean ±standard deviation. Different letters in the same column indicate a statistically significant difference (*p* <.05).

PEG films were thicker than glycerol films. Aimed at PEG films the thickness increased from 0.049 mm to 0.083 mm with increasing concentration of plasticizer, whereas in glycerol films thickness increased from 0.041 mm to 0.071 mm with increasing concentration of the plasticizer. This is caused by a higher molecular weight of PEG than glycerol. The molecular weight and interactions between incorporated components and polymers in film structure describe differences in density (Pelissari et al., 2013).

### 3.2 Mechanical properties

Mechanical properties are important features of edible films, especially at industrial levels, and are applied to determine the resistance of films to external influences and thus to assess the potential of their use as a packaging material (Mendes et al., 2017). Tensile strength (TS), elongation at break (E), and Young's modulus (YM) are measured for the fabricated USP films as shown in Table 1, concerning the plasticizer concentration levels. TS illustrates the film's mechanical resistance owing to cohesion forces among polymer chains, and E% calculates its flexibility, which is the capacity of a film to extend before breaking. YM is also an index to indicate the stiffness of the films.

Aimed at each film, the values of tensile strength and Young's modulus decreased with increasing plasticizer concentration. USP films plasticized with PEG has been demonstrated to have higher TS and YM values than glycerol films. TS of glycerol films and PEG‐films decreased from 20.25 to 5.91 MPa and 36.95 to 11.24 MPa, respectively, by increasing the proportion of plasticizer from 30% to 50% w/w (*p* ≤ .05). Increasing the amount of the plasticizer leads to a further increase in the intermolecular distance, increased mobility among the layers, and reduced interactions between the polysaccharide chains, which reduce the tensile strength and Young's modulus of the films (Zhang et al., 2006).

Glycerol reduced TS of films more effectively than PEG owing to hydroxyl groups, hydrophilic nature, and smaller molecular size that allows more and better placement of its molecules in the polymer chains. Thus, YM which indicates the stiffness of the films also decreased. According to Table 1, increasing the plasticizer concentration increased the E% values in both films. Glycerol films exhibited higher E% values than PEG‐films, and the films containing 50% glycerol and 30% PEG had the highest and lowest E% values, respectively. The increase in distance between polymer chains, increasing the flexibility of the films, and consequently, glycerol films showed more flexibility and E values than PEG films.

### 3.3 Moisture content and solubility

Moisture contents of plasticized USP films are provided in Table 2. Increasing the plasticizer concentration from 30% to 50% (w/w) significantly increased the moisture content of the USP films (*p* ≤.05). Glycerol films had more moisture content than PEG films. Because of the small size of glycerol molecules, they have more hydroxyl groups at the same concentration than PEG, thus binding to a greater number of water molecules in the polymer matrix (Antoniou et al., 2014).

**TABLE 2:** Effect of plasticizer types and concentration on moisture content, solubility, WVP, and OP of USP films

Film type	Moisture content (%)	Solubility (%)	WVP (g.mm^−1^s^−1^KPa−1 × 10^–11^)	OP (10^−8^cm^3^.cm/cm^2^.s.cmHg)
USP +30% Glycerol	19.96 ± 0.4^e^	49.4 ± 0.4^e^	1.34 ± 0.01^e^	1.49 ± 0.002^c^
USP +40% Glycerol	24.16 ± 0.2^c^	59.9 ± 0.2^c^	1.93 ± 0.01^c^	2.01 ± 0.001^b^
USP +50% Glycerol	32.86 ± 0.2^a^	67.2 ± 0.5^a^	2.32 ± 0.002^a^	2.98 ± 0.03^a^
USP +30% PEG 600	16.03 ± 0.1^f^	43.16 ± 0.3^f^	1.19 ± 0.009^f^	7.45 ± 0.002^f^
USP +40% PEG 600	20.9 ± 0.1^d^	54.13 ± 0.3^d^	1.73 ± 0.02^d^	7.84 ± 0.001^e^
USP +50% PEG 600	26.53 ± 0.4^b^	61.13 ± 0.3^b^	2.09 ± 0.005^b^	8.04 ± 0.04^d^

Note

Values are given as mean ±standard deviation. Different letters in the same column indicate a statistically significant difference (*p* < .05).

The results showed that with increasing the concentration of the plasticizer, the solubility increased significantly (*p* ≤.05) in both types of plasticizers (Table 2). Glycerol films showed higher water solubility than PEG‐films at the same concentrations. Plasticizers can increase film solubility by decreasing interactions between biopolymer molecules and help to retain more water in the film matrix. Because of glycerol`s small molecular size, the number of hydroxyl groups is higher in glycerol than PEG at the same concentrations. Thus, the attracted water into the polymer matrix and the hydrophilicity increased.

### 3.4 Water vapor permeability

Barrier properties in packaging films play an important role in eliminating or reducing the water, oxygen, carbon dioxide, or aroma exchanged among food components and the storage environment (Qi et al., 2016). The WVPs of films containing various plasticizer concentrations are shown in Table 2. The results showed that with increasing the concentration of both types of plasticizers, the vapor permeability of the films increased. When films are exposed to water vapor, the hydrophilic structure of the film absorbs water and then the water molecules gradually move through the film and evaporate from there (Sadeghi et al., 2018).

It has been shown that water vapor permeability depends on the type and concentration of the plasticizer. According to Fick's and Henry's laws, water vapor permeability is a function of both solubility and diffusion processes. Therefore, plasticizers not only increase the water solubility in the polymer matrix, but also increase the mobility of the chains, soften the polymer, and increase the diffusion coefficient [13]. The results showed that the water vapor permeability in glycerol films is higher than that in PEG films, which can be justified due to the higher solubility of glycerol films than PEG films. Haq et al. (2014), Gheribi et al. (2018), and Wittaya (2013) concluded that glycerol films have more water vapor permeability than PEG films and have less water vapor barrier properties.

### 3.5 Oxygen permeability

Permeability to ambient vapors and gases affects the mechanical properties of packaging as well as the reduction in volatile food compounds such as flavorings, or the transfer of unpleasant environmental compounds through gases such as oxygen and carbon dioxide to the packaging materials. Therefore, in most cases, low permeability to gases is a positive and important feature in maintaining food quality (Sánchez et al., 2011).

The effect of plasticizer on oxygen permeability (OP) of USP films is shown in Table 2. By increasing the concentration of oxygen, permeability to oxygen increased significantly, and the highest and lowest OP was found in films plasticized with 50% glycerol and films plasticized with 30% PEG, respectively. According to Srinivasa et al. (2007), plasticizers decrease the resistance of the films to oxygen transfer by increasing the mobility of the polymer chains. It has also been reported that hydrophilic films and coatings especially polysaccharide‐based films, are generally good barriers to oxygen transport (Wittaya, 2013).

### 3.6 Transparency

Transparency of the USP films is reported in Table 3. The results showed that with increasing concentration in both plasticizers, the transparency of the films decreased significantly. Film plasticized with 30% PEG and 50% glycerol showed the highest (4.53) and lowest (2.66) values of transparency, respectively. These results are attributed to the increase in intermolecular distance and increased mobility between polymer chains. On the other hand, by comparing the results, it was found that glycerol films were less transparent than PEG‐films. Differences in the transparency of films may be owing to differences in molecular weight, composition, size, nature, and the functional properties of plasticizers that can prevent light transmission from the films (Orliac et al., 2003).

**TABLE 3:** Effect of plasticizer type and concentration on L*, a*. b* and transparency of USP films

Film type	L*	a*	b*	Transparency
USP +30% Glycerol	99.18 ± 0.19^a^	−0.98 ± 0.03^a^	4.23 ± 0.28^a^	4.24 ± 0.35^a^
USP +40% Glycerol	99.01 ± 0.34^a^	−1.09 ± 0.12^a^	4.27 ± 0.19^a^	2.98 ± 0.07^bc^
USP +50% Glycerol	98.98 ± 0.25^a^	−1.02 ± 0.16^a^	4.37 ± 0.33^a^	2.66 ± 0.19^c^
USP +30% PEG 600	99.09 ± 0.15^a^	−1.03 ± 0.08^a^	4.20 ± 0.21^a^	4.53 ± 0.13^d^
USP+40% PEG 600	99.05 ± 0.54^a^	−1.02 ± 0.18^a^	4.22 ± 0.14^a^	3.33 ± 0.08^e^
USP +50% PEG 600	99.02 ± 0.22^a^	−1.07 ± 0.14^a^	4.25 ± 0.09^a^	3.01 ± 0.08^be^

Note

Values are given as mean ±standard deviation. Different letters in the same column indicate a statistically significant difference (*p* < .05).

### 3.7 Color measurement

Visual properties are required to demonstrate the ability to use films and coatings, as these properties affect the appearance of the coated product, which is an important quality factor (Seyedi et al., 2014). Among these features, color properties are of particular importance because they directly affect consumer acceptance. In general, the desirable features in packaging films and coatings are high transparency and lightness (Razavi et al., 2015).

The results of the USP films' color characteristics are presented in Table 3. According to Table 3, with increasing the concentration of plasticizer, the amount of lightness (L*) showed a slight decrease; these changes and the trend were non‐significant (*p* >.05). The values of redness (a*) was not influenced by the plasticizer concentration significantly (*p* >.05). The values of yellowness (b*) in the present research increased by increasing plasticizer concentration, nonetheless these changes were non‐significant (*p* >.05).

### 3.8 FTIR analysis

**FIGURE 1:**
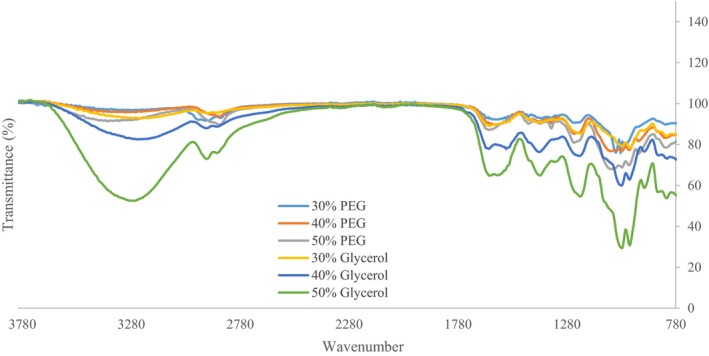
Effect of plasticizer type and concentration on the FTIR spectrum of USP films

Figure 1 shows the FTIR spectrum of the USP films using two plasticizers. The broadband ranging from 3240 cm^−1^ to 3410 cm^−1^ refers to the stretch of bonded hydroxyl (O‐H) and bound water. Film plasticized with 50% glycerol had the highest absorption i.e. the lowest transmittance in this band with a wavenumber of 3,265.1 cm^−1^. This group of hydroxyl agents can be free or in the form of hydrogen bonds. These hydrogen bonds can generally exist in three types: hydrogen bonds present within the polymer structure, hydrogen bonds present within the structure of the plasticizer, and the bonds that form between the plasticizer and the polymer structure. It has been reported that this peak can also show the rate of adsorption of water molecules in the film structure, which can be used to obtain information about the hydrophilicity of the polymer (Antoniou et al., 2014; Zhang et al., 2016). Concerning both plasticizers, with increasing concentration, water absorption and the number of hydroxyl groups increased. Moreover, glycerol films had higher adsorption than PEG films, indicating that these films absorb more water and therefore have more hydroxyl groups in their structure.

The broadband around 2800–3000cm^−1^ is attributed to C‐H stretching vibration. Films plasticized with 50% glycerol had the highest absorption at wavenumber 2,933.53 cm^−1^. This can be attributed to the smaller size of glycerol molecules, which causes them to penetrate more into the polymer structure and form more bonds. The band from 1,500 to 1700cm^−1^ describes the stretch of carbonyl groups (C = O) groups and carboxyl ion stretching bands (COO^‐^) (Velazquez et al., 2003; Zhang et al., 2016). The peak at 1400 cm^−1^ and the peaks around 1000–1400 cm^−1^ attributed to asymmetric stretching vibrations of the COO bonds and the symmetric tensile vibrations of the COO group, respectively.

### 3.9 Scanning electron microscopy (SEM)

**FIGURE 2:**
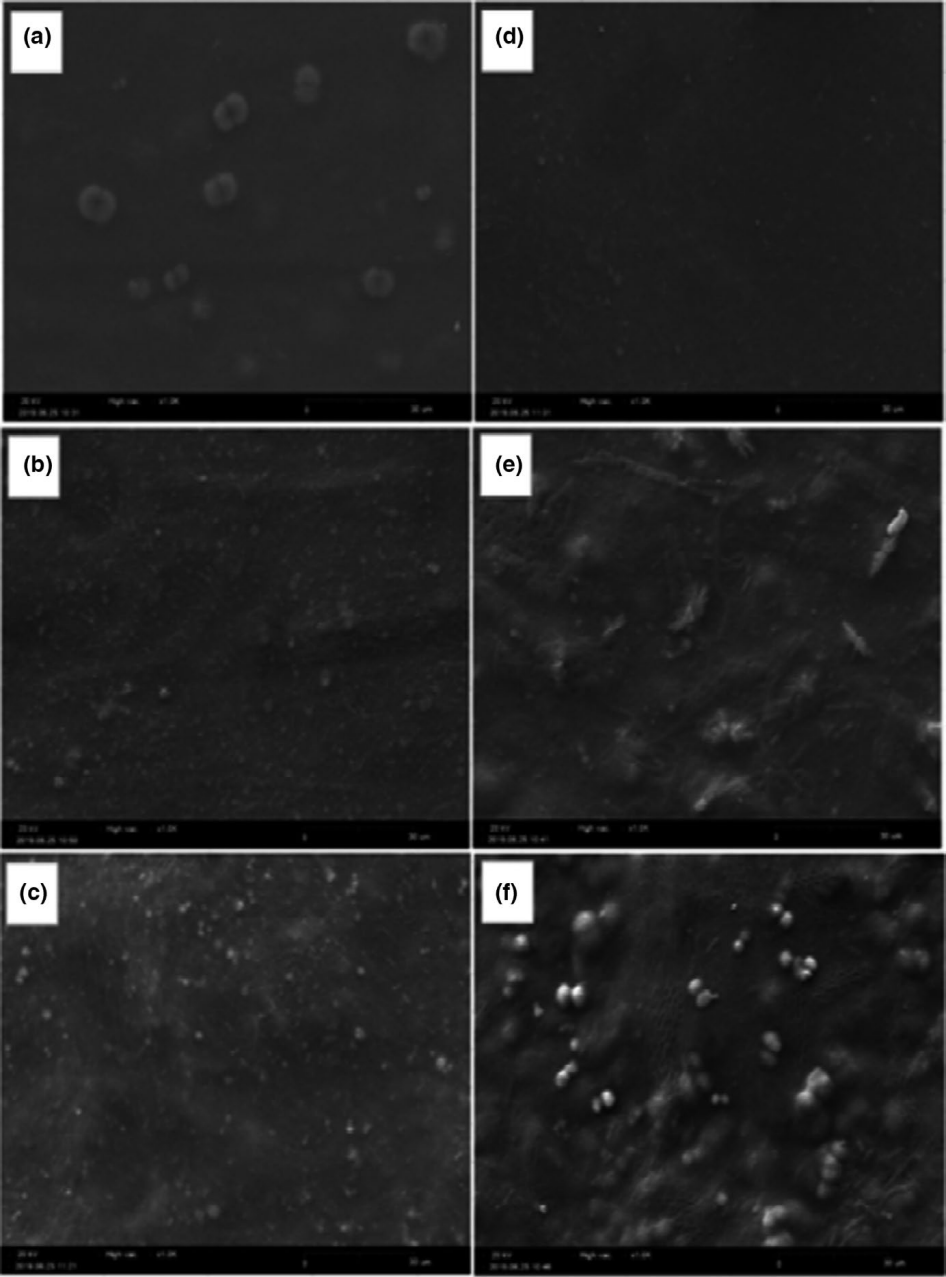
Scanning electron micrograph of USP films (magnification 1,000). (a) film plasticized by 30% glycerol, (b) 40% glycerol, (c) 50% glycerol, (d) 30% PEG, (e) 40% PEG, and (f) 50% PEG

*SEM* analysis was applied to observe the morphology of films. Figure 2 shows that the surface of the films was not broken or cracked and it was free of bubbles. Comparing the images together, it was evident that glycerol films have a smoother and more uniform surface than PEG films. PEG films had a rougher surface than glycerol films, which may indicate the larger size of PEG molecules than that of glycerol; consequently, these molecules are less able to penetrate the polymer structure.

## 4 CONCLUSION

The results of this research showed that USP could be a new source for edible film production, nevertheless the mechanical, optical, physical, and barrier properties of the films were a function of the type and concentration of plasticizer. The results indicated that by increasing plasticizer concentration, thickness, moisture, solubility, elongation at to break, WVP, and OP values of the films increased, and, in return, TS, YM, and transparency values of the films decreased. The brightness, redness, and yellowness of the films did not change significantly with increasing the concentration of plasticizers. *SEM* analysis correspondingly showed that all of USP films had a surface free of cracks, fractures, and bubbles. It was found that film plasticized with 30% PEG had the lowest moisture content and solubility and the highest TS, YM, and transparency.

## CONFLICT OF INTEREST

The authors declare that they do not have any conflict of interest.

## AUTHOR CONTRIBUTION

**Mohammad Davoudi:** Data curation (equal); Investigation (equal); Writing‐original draft (equal).

## ETHICAL APPROVAL

Ethical Review: This study does not involve any human or animal testing. Informed Consent: Written informed consent was obtained from all study participants.
